# Differentially localized protein identification for breast cancer based on deep learning in immunohistochemical images

**DOI:** 10.1038/s42003-024-06548-0

**Published:** 2024-08-02

**Authors:** Zihan Zhang, Lei Fu, Bei Yun, Xu Wang, Xiaoxi Wang, Yifan Wu, Junjie Lv, Lina Chen, Wan Li

**Affiliations:** College of Bioinformatics Science and Technology, Harbin Medical University, Harbin, 150000 China

**Keywords:** Machine learning, Protein analysis

## Abstract

The mislocalization of proteins leads to breast cancer, one of the world’s most prevalent cancers, which can be identified from immunohistochemical images. Here, based on the deep learning framework, location prediction models were constructed using the features of breast immunohistochemical images. Ultimately, six differentially localized proteins that with stable differentially predictive localization, maximum localization differences, and whose predicted results are not affected by removing a single image are obtained (CCNT1, NSUN5, PRPF4, RECQL4, UTP6, ZNF500). Further verification reveals that these proteins are not differentially expressed, but are closely associated with breast cancer and have great classification performance. Potential mechanism analysis shows that their co-expressed or co-located proteins and RNAs may affect their localization, leading to changes in interactions and functions that further causes breast cancer. They have the potential to help shed light on the molecular mechanisms of breast cancer and provide assistance for its early diagnosis and treatment.

## Abstract (web summary)

Six differentially localized proteins are obtained through protein subcellular localization prediction models constructed by deep learning in immunohistochemical images, providing support for breast cancer early diagnosis and treatment.

## Introduction

Breast cancer is one of the most prevalent cancers in the world, with 2.3 million women diagnosed and 685,000 deaths globally in 2020. With the improvement of the level of diagnosis and treatment, breast cancer treatment can achieve survival probabilities of 90% or higher, particularly when the disease is identified early^1^. The current clinical testing and treatment decisions are usually based on information at the protein level^2^. It is of great significance to analyze the proteome data of breast cancer.

In human cells, many proteins exhibit highly complex subcellular distribution patterns. In order to perform cell functions, some proteins are located in multiple organelle^3^, and protein subcellular locations also show diversity among different tissues, cell types, and cell conditions^4^. The subcellular localization of proteins can also provide important clues for understanding protein functions in cells. Protein can only function at the correct location of the cell compartments, and mislocalization positioning of proteins can lead to diseases, including cancer^5^. For example, in normal tissues, the protein FOXO3 is mainly located in the nucleus, while the wrong positioning of the protein FOXO3 in the cytoplasm has been proved to be related to the low survival rate of human breast cancer^6^. Proteins that change position do not always change expression, but they also play an important role in disease processes, which can be obtained through subcellular localization change analysis. Further, discovering proteins that relocate in the disease state may play an important role in changes driving disease, and such changes would go undetected by measuring only expression^4^.

In previous studies, the subcellular localization of proteins was mostly predicted through tools constructed based on amino acid sequence analysis^7–9^. However, due to the limited protein subcellular localization information carried by protein sequences, sequence-based predictors struggle to achieve a satisfactory accuracy. In recent years, microscopic images captured at the single-cell level have accumulated rapidly, which makes the prediction of protein subcellular localization more accurate and allows tissue-specific studies^10^. The microscopic imaging of cellular and subcellular structures belongs among the most widely used techniques in biomedical research and clinical diagnosis^11^. Immunohistochemical (IHC) images, which are widely used as the gold standard for cancer diagnosis, provide expression and localization information of specific proteins in complete tissues, making it an important data source to identify the subcellular localization of proteins. Compared with amino acid sequence one-dimensional, IHC image can not only intuitively display the complex pattern of specific proteins from the tissue level to the cell level, but also display the protein translocation associated with disease, which plays an irreplaceable role in cancer analysis^12^. In particular, The tissue Atlas of Human Protein Atlas (HPA; https://www.proteinatlas.org/; version: 23.0) database contains a large number of IHC images, covers the distribution and expression of more than 26000 human proteins in 44 human normal tissues and 17 tumor tissues, which provides important resources for proteomics studies^13^.

With the rapid improvement in the quantity and quality of microscopic images, many image-based automatic localization prediction methods have been developed. Deep learning is a subfield of machine learning, renowned for its ability to correctly classify and segment images, which has achieved outstanding results in various fields, especially in the biomedical industry^14^. Convolutional neural network is one of the representative algorithms of deep learning, and the combination of image processing and convolutional neural networks is one of the successful examples of deep learning in recent years^15^. Attention mechanism is a method that mimics the human visual and cognitive system. By introducing attention mechanism, neural networks can automatically learn and selectively focus on important information in the input, improving the performance and generalization ability of the model^16^. Attention mechanism is an algorithm that mimics human visual and cognitive systems. By introducing attention mechanism, neural networks can automatically learn and selectively focus on important information in the input, improving the performance and generalization ability of the model^16^. Currently, there are only a few studies using deep learning and this is one of the pilot studies on utilizing deep learning approaches to predict protein localization.

In this study, convolutional neural network and attention mechanism were combined and models based on deep learning framework were built to analyze the features of IHC images of breast tissues and identify differentially localized proteins in breast cancer. Ultimately, six differentially localized proteins were obtained (CCNT1, NSUN5, PRPF4, RECQL4, UTP6, ZNF500). Further verification showed that these proteins were closely related to breast cancer and could well distinguish between cancer and normal samples. Potential mechanisms of these proteins and the effect of their localization changes on the occurrence of breast cancer were analyzed, which will contribute to the detection and treatment of breast cancer, as well as the further study of protein mislocalization and cancer molecular mechanism.

## Results

### Protein localization prediction models

**Fig. 1:**
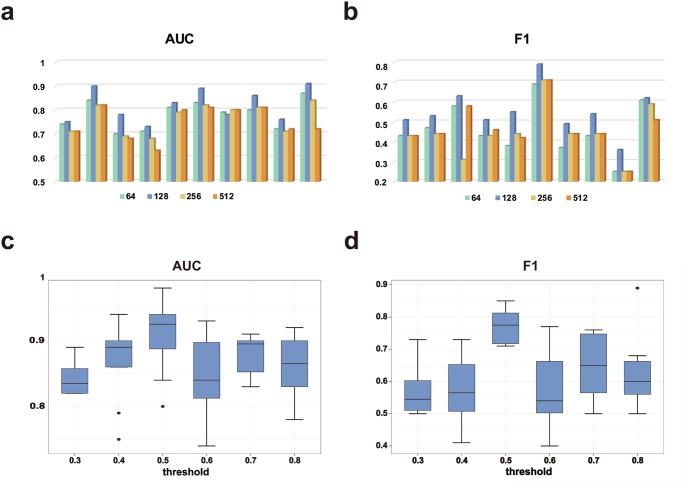
Predicted performance of models. **a** AUC value of the predicted results of features in 64, 128, 256, and 512 dimensions (*n* = 33); (**b**) F1 score of the predicted results of features in 64, 128, 256, and 512 dimensions. **c** AUC value of the predicted results of 6 thresholds (*n* = 33); (**d**) F1 score of the predicted results of 6 thresholds.

Based on the deep learning framework, location prediction models were constructed (see “Materials and Methods” for details). In order to determine the optimal feature dimension, the features of IHC images in 64, 128, 256, and 512 dimensions were extracted from the four convolutional layers of the ResNet_18 to construct models, respectively. Image features of normal tissue were used for ten-fold cross validation to predict the localization of proteins, and the Area Under Receiver Operating Characteristic Curve (AUC) value and F1 score of the predicted results were calculated to evaluate the models. It was found that the models constructed with 128 dimensional feature vectors yielded the best results in most cases (Fig. 1a, b). In particular, during the model training process, 90% of the proteins were randomly selected to train the models through the 10-fold cross validation, and the remaining 10% were used as an independent validation set for the final performance evaluation to ensure the confidence of the models. The result AUC value was 0.85 and F1 score was 0.65, which was similar to the results of 10-fold cross validation and could be considered that our models were not overfitting.

At the same time, the prediction probability threshold was also trained. The classification performance of 6 thresholds were compared (0.3, 0.4, 0.5, 0.6, 0.7, 0.8) and it was found that when the threshold was set to 0.5, both AUC value and F1 scores were higher. Here we only provided the results of 128 dimensional features (Fig. 1c, d).

Optimal parameters, 128 dimension and 0.5 threshold, were selected as the final model parameters for subsequent analysis. In order to obtain more stable results, the 70% and 90% sampling methods were adopted to obtain two sets of models, each with 10 models. Among them, the average AUC value of the prediction results of the 90% random sampling models was 0.91 and the average F1 score was 0.76, while the average AUC value of 70% was 0.92, and the average F1 score was 0.81 (The detailed results of all models were presented in Supplementary Table 1). Both sampling methods achieved good AUC value and F1 score, which indicating robust results of our models.

In addition, two existing methods for protein subcellular localization prediction based on convolutional neural networks, AnnoFly and Imploc, were used. To ensure the accuracy of the results, the same data and sampling methods were used for the three models. The AUC value and F1 values were calculated and it was found that our models achieved better predictive effectiveness. Our model outperforms other models in the same background, demonstrating the accuracy and usability of our model (Table 1).

**Table 1:** Model effectiveness evaluation

	90%sample		70%sample	
	AUC	F1	AUC	F1
AnnoFly	0.73	0.42	0.74	0.44
Imploc	0.82	0.56	0.86	0.64
Protein localization prediction model	0.92	0.76	0.92	0.81

### Differentially localized proteins

**Fig. 2:**
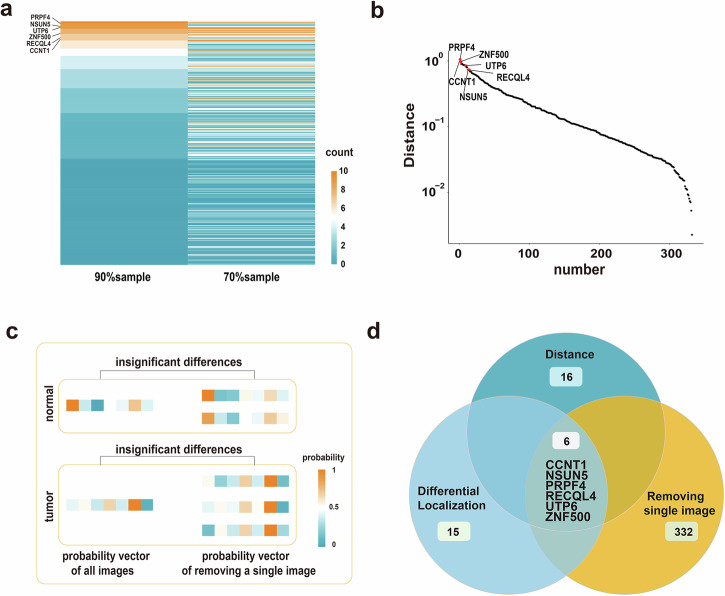
Differentially localized proteins. **a** The number of times that the predicted location of each protein was different in the 90% and 70% random sampling methods results. **b** The distribution of the distance between the predicted results of proteins under disease and normal conditions. **c** The comparison of predicted results between removing a single image and all images (**d**) The differentially localized proteins obtained from the intersection of three steps.

According to the constructed subcellular localization prediction models, the differentially localized proteins were identified. Three steps were taken (see “Materials and Methods” for details) and proteins with different prediction results in more than 75% of the cases, the top 5% largest localization difference between disease and normal states, and no significant impact by removing a single image were obtained (Fig. 2a–c). Six breast cancer differentially localized proteins were got: CCNT1, NSUN5, PRPF4, RECQL4, UTP6, ZNF500 (Fig. 2d).

### Differentially localized proteins verification

**Fig. 3:**
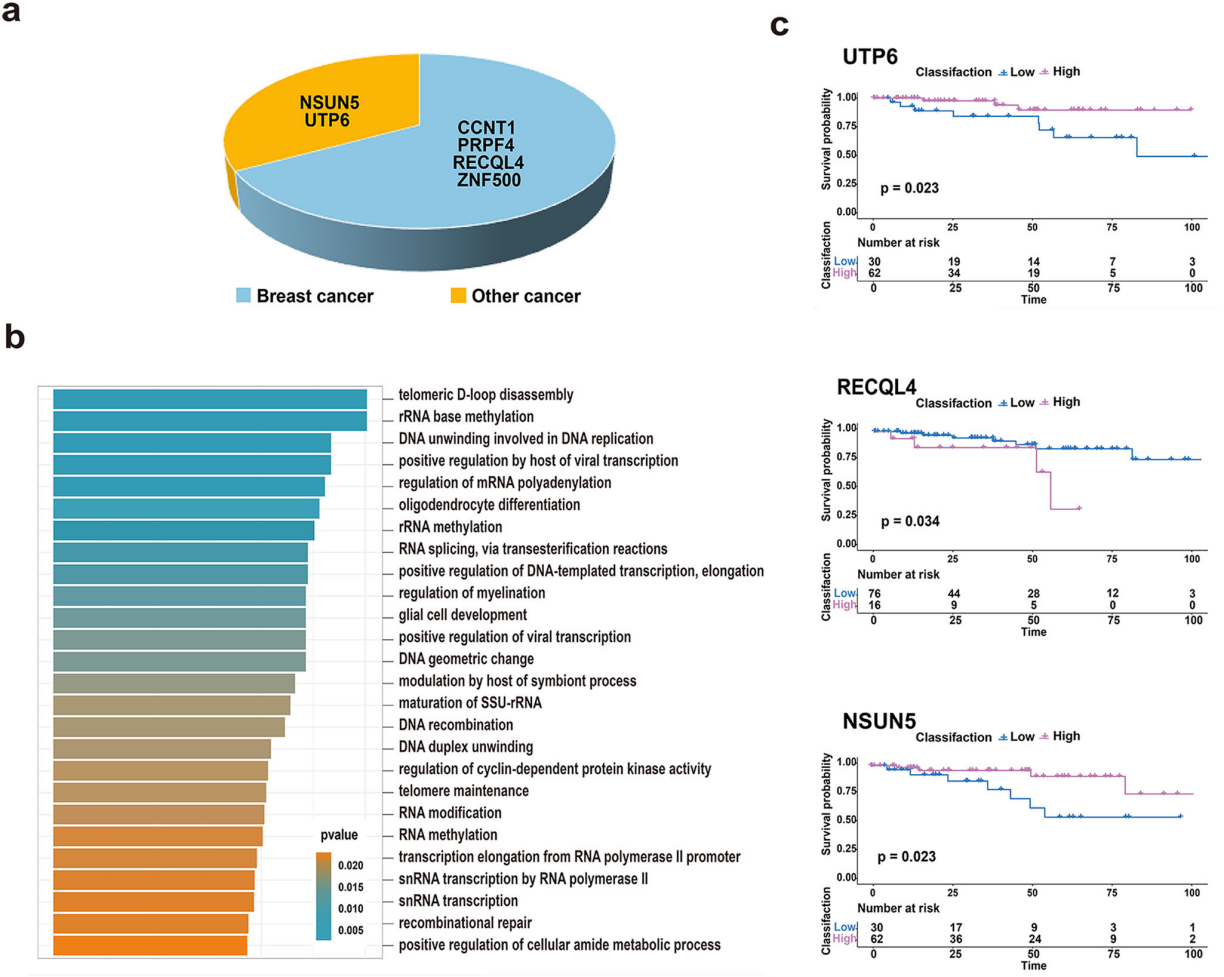
Differentially localized proteins verification. **a** Literature validation of the relationship between protein and breast cancer. **b** Results of functional analysis of the six differentially located proteins. **c** Survival curves of the proteins (*n* = 92).

To analyze the association between differentially localized proteins and breast cancer, literature in the PubMed database were browsed. It was found that four of the six proteins are closely related to breast cancer (CCNT1, PRPF4, RECQL4, ZNF500) (Fig. 3a). CCNT1 affects the transcriptional activity of breast cancer by participating in the estrogen receptor alpha regulates transcription process^17^, while it is also down regulated during the treatment of breast cancer through targeting polyamine biosynthesis caused by RNAi^18^. PRPF4 affects the growth, migration, invasion, and apoptosis of breast cancer cells through p38 MAPK signaling pathway, which is a therapeutic target for breast cancer^19^. RECQL4 locates at the common amplification site in sporadic breast cancer and plays a carcinogenic role in breast tumorigenesis^20^. ZNF500 abolishes breast cancer proliferation and sensitizes chemotherapy by stabilizing P53 via competing with MDM2^21^. NSUN5 and UTP6 play important roles in cancers such as colorectal cancer and clear cell renal cell carcinoma^22–24^, and UTP6 was discussed in breast tumors core needle biopsies before^25^. These proteins might also play an important role in the progression of breast cancer.

Based on the enrichr function in the R package “enrichR”, the functional analysis was performed. The functional of six differentially located proteins was analyzed and 97 significantly enriched pathways and biological functions were obtained (Fig. 3b), which mainly focused on RNA splicing, maturation of SSU-rRNA, regulation of regulation of cyclin-dependent protein kinase activity, RNA modification, RNA methylation, 7SK snRNA binding, snoRNA binding and other aspects. CCNT1 was enriched in the 7SK snRNA binding process, 7SK snRNA involved in the regulation of positive transcription elongation factor b on the epithelial–mesenchymal transition, which is important for breast cancer progression^26^. UTP6 was enriched in the process of snoRNA binding, while snoRNA activated PARP-1 ADPRylates DDX21 in BRCA1/2-wild-type breast cancer cells to promote enhanced ribosome biogenesis and cell proliferation^27^. RECQL4, UTP6, CCNT1, and NSUN5 were enriched in the regulation process of cyclin dependent protein kinase activity, Cyclin D1 is one of the effectors of tumor gene Neu and Ras causing malignant transformation of cells in breast cancer^28^. ZNF500 participated in transcriptional regulation of RNA polymerasII. Inhibiting Ribosome biogenesis with RNA transcription as the initial step has become a promising strategy in breast cancer treatment^29^.

Due to the lack of survival data of the samples in PDC000120, only PDC000173 was analyzed in this part. Among them, ZNF500 did not have expression data, so survival analysis was performed on the other 5 proteins. Based on the results of survival analysis (*p* < 0.05), it was found that the results of UTP6, NSUN5 and RECQL4 proteins were more relevant to the prognosis of breast cancer, while the results of CCNT1 and PRPF4 were not (Fig. 3c). These three proteins could be used as prognostic markers, which was helpful to the treatment of breast cancer.

The above verification suggested that the proteins we found were closely related to breast cancer, meaning that their location changes play an important role in the occurrence of the cancer.

### Classification effectiveness

**Fig. 4:**
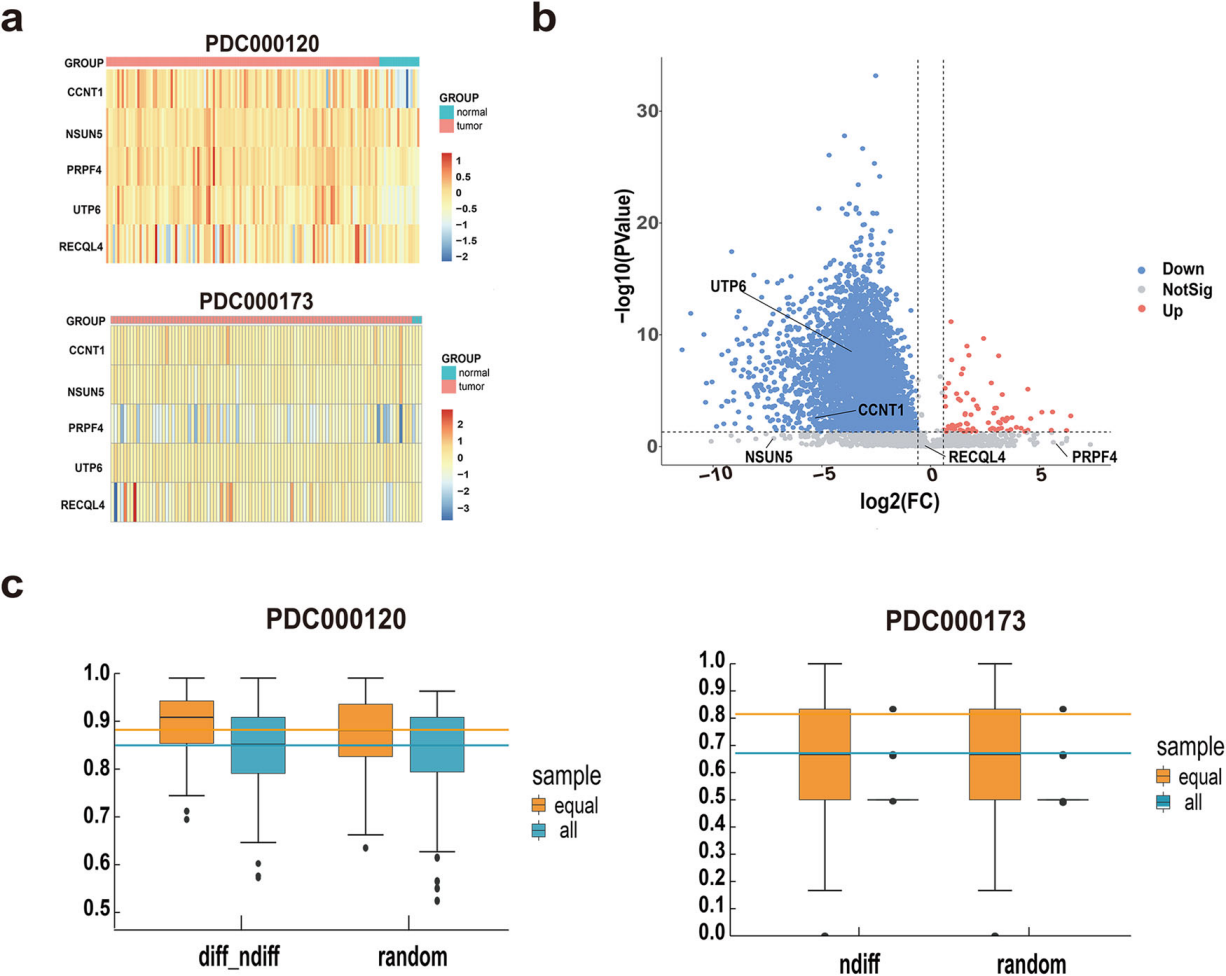
Classification effectiveness. **a** Heatmap of the expression of differentially localized protein in two protein expression profiles. **b** Differential expression of differentially expressed proteins in profile PDC000120. **c** Classification results of two protein expression profiles, PDC000120 (*n* = 141) and PDC000173 (*n* = 95).

Based on the two protein expression profiles of breast tissue downloaded from the CPTAC database, differentially expressed proteins were identified, respectively. Among the 6 proteins we obtained, there were no expression values of ZNF500 in either data set, so only the remaining 5 proteins were analyzed. Among them, the profile PDC000120 obtained 4060 differentially expressed proteins, of which UTP6 and CCNT1 were differentially downregulated proteins, while the other three were not (Fig. 4a, b). Meanwhile, 276 differentially expressed proteins were obtained in PDC000173, but none of the 5 proteins we obtained were there. Although they were not differentially expressed proteins, literature and enrichment results have demonstrated their relevance to breast cancer, indicating that subcellular localization analysis could yield results that differential expression analysis cannot obtain.

A classifier was constructed based on random forest to classify the samples in the profiles, and its classification performance was evaluated through the the Leave One Out Cross Validation method. Then, an equal number of non-differentially localized proteins were randomly selected to construct classifiers. Due to the difference in the number of normal and cancer samples in the expression profile, in order to eliminate the impact of uneven sample size, the same number of normal samples were randomly selected from cancer samples for classification. Meanwhile, as the classifier was constructed based on expression values, the impact of differentially expressed proteins on the classification results was also considered. In the profile PDC000120, since two differentially expressed proteins were differentially localized proteins, in addition to randomly sampling all proteins, equal number of differentially and non-differentially expressed proteins were randomly selected for analysis. It was found that the AUC values of differentially localized proteins were similar to those predicted by other proteins, and some results of non-differentially localized proteins had higher AUC values than those we obtained. However, T-test analysis showed that the difference between the two types of results was not significant. In the PDC000173 expression profile, there were no differentially expressed proteins. Randomly selecting all proteins and non-differentially expressed proteins for analysis, it was found that the predicted results of differentially localized proteins were superior to those of other proteins. It proved that the differentially localized protein we obtained can effectively distinguish cancer from normal samples (Fig. 4c).

### Breast cancer potential mechanisms

**Fig. 5:**
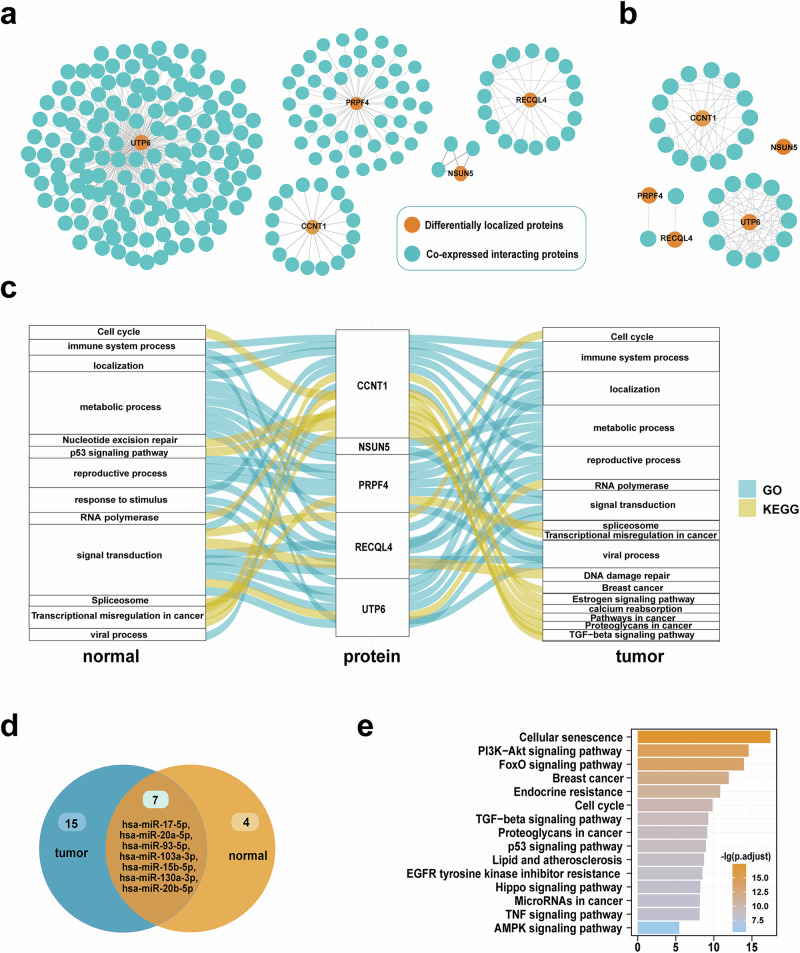
Potential mechanisms analysis of differentially localized proteins. **a** Modules composed of differentially localized proteins and co-expressed interacting proteins under tumor conditions. **b** Modules composed of differentially localized proteins and co-expressed interacting proteins under normal conditions. **c** Functional changes of differentially localized proteins between tumor and normal conditions. **d** Interaction ncRNAs of differentially localized proteins. **e** Functional enrichment of Interaction ncRNAs.

Interacting proteins in normal and cancer samples were studied separately. Proteins whose correlation coefficient >0.3 and *P* < 0.05 and with a String database interaction score of higher than 700 were extracted as the significantly co-expressed interacting proteins of differentially localized proteins. Due to the lack of expression data for ZNF500, only the correlation and interaction relationships of the remaining 5 proteins were discussed, and ultimately two sets of interacting proteins were obtained and interaction modules were constructed (Supplementary Table 2; Fig. 5a, b).

To analyze functions of the proteins obtained, enrichment analysis was done through GO and KEGG methods (Fig. 5c). It was found that in both normal and disease conditions, proteins were enriched in immune, transcription, metabolism, localization, signal transduction, and other basic pathways. However, under cancer conditions, differentially localized proteins and their interacting molecules were more enriched in biological functions and pathways related to cancer and breast cancer. The proteins specifically enriched to breast cancer, estrogen signaling pathway, the TGF-β signaling pathway, and so on, which had been proved to play an important role in breast cancer. The effect of estrogen is mainly achieved by binding to estrogen receptor, whose imbalance can promote the excessive proliferation of breast cells or block the apoptosis of breast cells, thus leading to the occurrence of breast cancer^30^. TGF-β signaling pathway mediates the regulation of epithelial mesenchymal transformation, which plays an important role in the occurrence and metastasis of breast cancer^31^. This indicated that changes in the localization and regulation of the proteins we obtained may affect the occurrence of breast cancer. The change of protein interactions in the modules might affect the localization of the proteins we identified, thus changing their functions, and participating in the occurrence of breast cancer.

Based on RNA protein interaction data in the RNA Interactome Database (RNAInter; http://www.rna-society.org/rnainter3/home.html; accessed on 25 March 2023) and RNA localization data in the RNALocate (http://www.rna-society.org/rnalocate/; accessed on 25 March 2023) database, the non-coding RNAs interacting with differentially localized proteins were analyzed. It was consistent with the subcellular localization in breast cancer celllines in the SUBCELL BARCODE database (https://www.subcellbarcode.org/; accessed on 13 June 2022). The ncRNAs co-located with differentially localized proteins in both conditions were extracted, and 11 and 22 ncRNAs were obtained, respectively. Among them, 7 miRNAs had the same localization as differentially localized proteins in both cancer and normal conditions, which might undergo localization changes together with the proteins (Fig. 5d). Through searching the KEGG database, five of the seven miRNAs were found in the MicroRNAs in cancer pathway and hsa-miR-103a-3p was in the breast cancer related areas. Enrichment analysis of the miRNAs in RNAenrich database (http://idrblab.cn/rnaenrich/; accessed on 11 September 2023) found that seven RNAs were enriched in 124 KEGG pathways, including breast cancer pathway, PI3K-Akt signaling pathway, FoxO signaling pathway, etc (Fig. 5e). Among them, the upregulation of PI3K-AKT signaling pathway is a potential indicator of the lack of response of stage II/III breast cancer patients to NAC, which lays a foundation for revealing the underlying pathogenesis^32^. Loss of FoxO3 function can enhance the metastatic potential of breast cancer cells in vivo and in vitro^33^. Browsing the CancerMIRNome database^34^ (http://bioinfo.jialab-ucr.org/CancerMIRNome/; accessed on 25 June 2022), it was found that 4 of the 7 miRNAs enriched in the breast cancer pathway (*hsa-miR-15b-5p*, *hsa-miR-20a-5p*, *hsa-miR-93-5p*, *hsa-miR-20b-5p*), 5 of the 7 miRNAs enriched in the TGF-β signaling pathway (*hsa-miR-17-5p*, *hsa-miR-20a-5p*, *hsa-miR-93-5p*, *hsa-miR-130a-3p*, *hsa-miR-20b-5p*) and *hsa-miR-103a-3p* enriched in the AMP-activated protein kinase signaling pathway. These results indicated that the miRNAs we obtained were closely related to breast cancer, and might affect the localization of differentially localized proteins, which causing changes in protein functions, thereby affecting the occurrence of breast cancer.

**Fig. 6:**
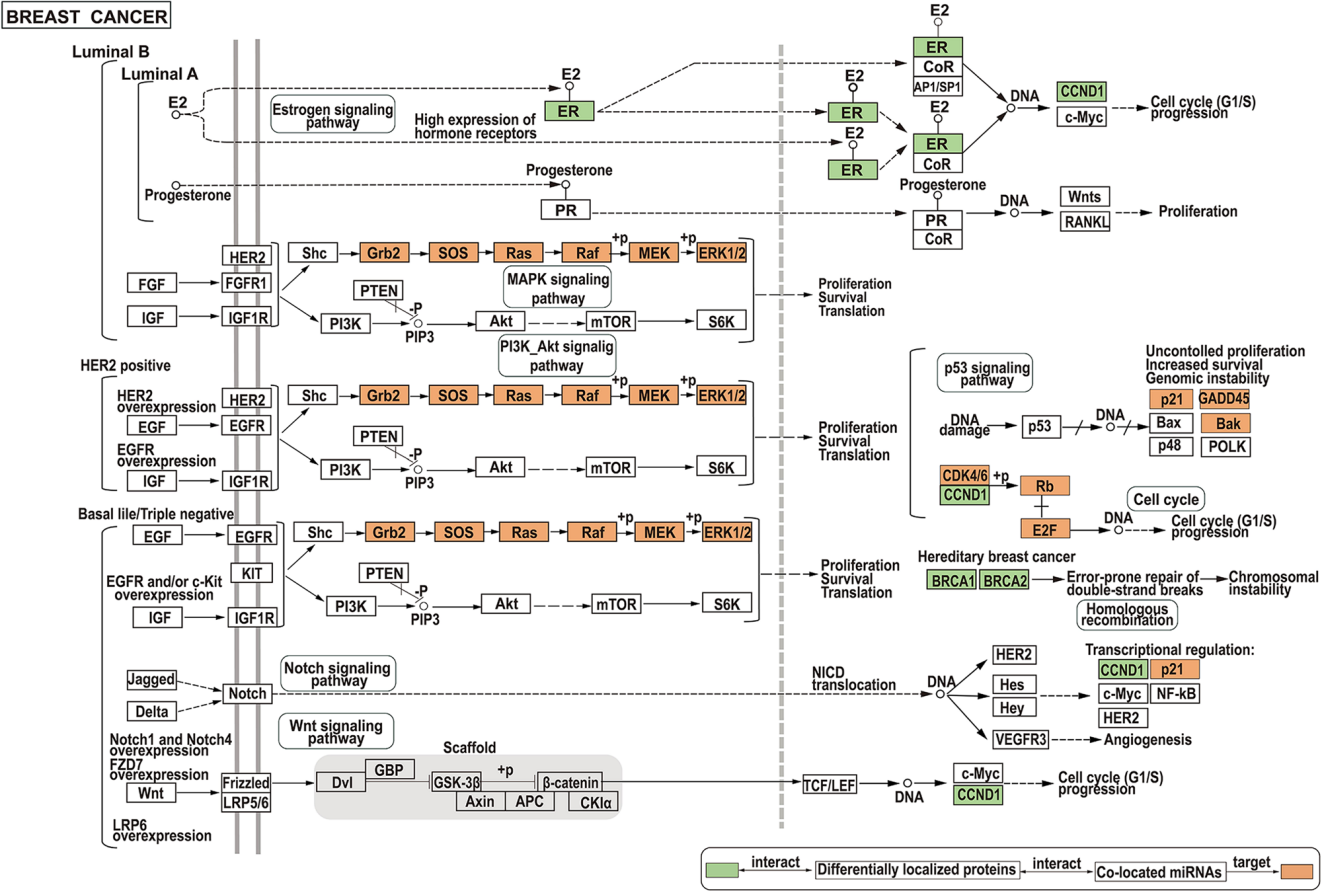
Breast cancer pathway^48^. The green rectangle represents proteins interact with differentially localized proteins and the orange rectangle represents potential targets for miRNAs we obtained.

In addition, to further investigate the molecular mechanism of breast cancer, the breast cancer pathway in the KEGG database was analyzed. It was found that multiple proteins in the pathway, such as ER and CCND1, interact with differentially localized proteins. Then, based on the target information of miRNA in the starBase database^35^ (https://rnasysu.com/encori/; accessed on 11 September 2023), it was found that the co-located miRNAs that interact with the proteins can also target the molecules in the pathway (Fig. 6). The results indicated that the proteins we got were closely related to breast cancer.

## Discussion

Based on the deep learning model, the features of breast IHC images were analyzed and models were constructed to identify the differentially located proteins of breast cancer. Six differentially localized proteins were obtained. Through verification, it was found that these proteins were not differentially expressed, but could well distinguish between cancer and normal samples, indicating that they were closely related to breast cancer. In addition, their potential mechanisms were also analyzed and the co-expressed or co-located proteins and RNAs were obtained. It was found that the changes in their subcellular localization led to changes in interactions and functions, which in turn affected the occurrence of breast cancer.

**Fig. 7:**
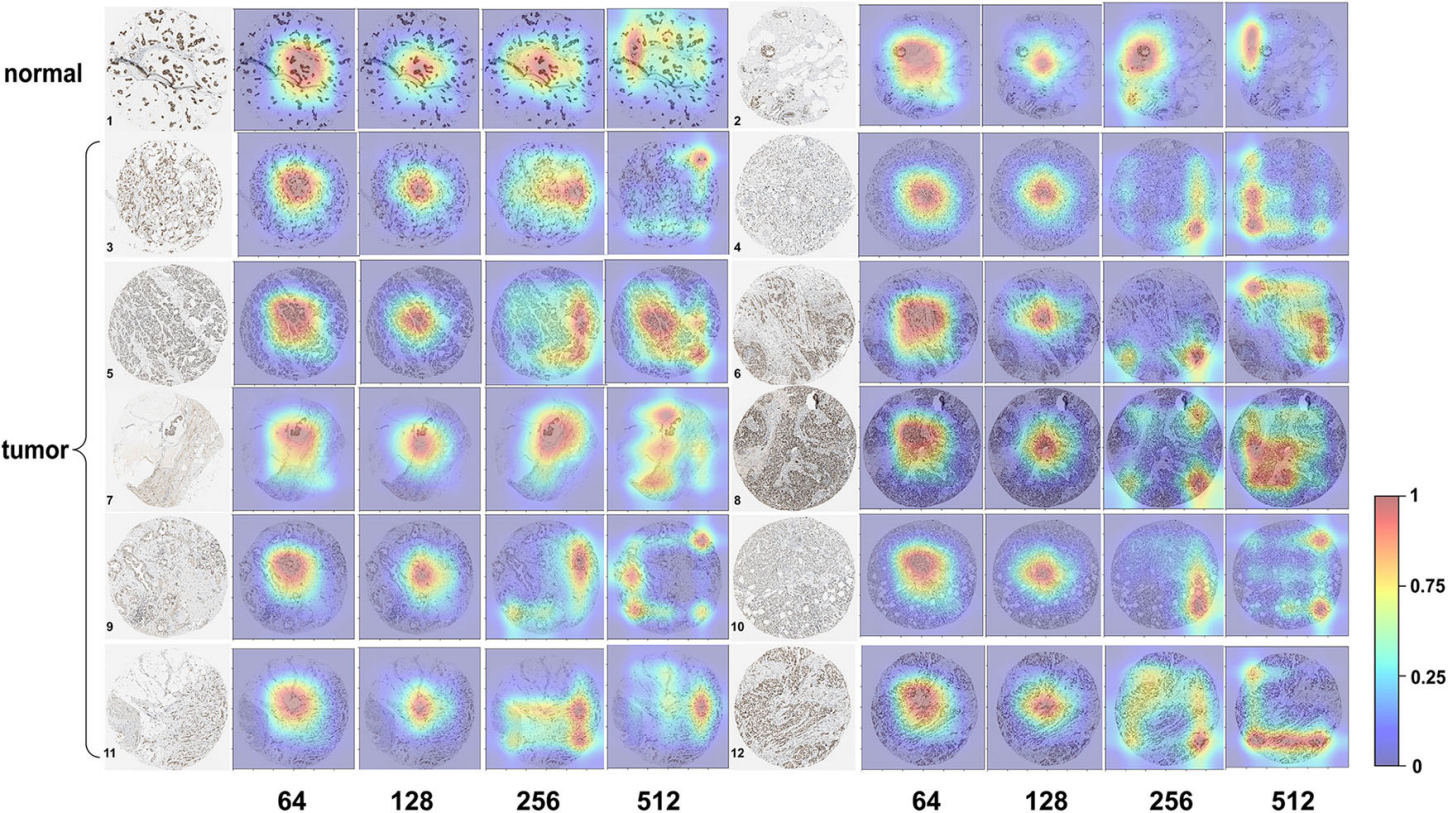
IHC image and Grad-CAM analysis results of CCNT1. The first two images are IHC images of normal breast tissue, while the others are images of tumor tissue. Each IHC image corresponds to four heatmaps of key regions for feature in different dimensions, corresponding to 64, 128, 256, and 512 dimensions.

It is worth noting that the interpretability of deep learning models is crucial in the medical context. Gradient-weighted Class Activation Mapping (Grad-CAM) was used to explore the interpretability of the model and provide a detailed description of its effectiveness. Grad-CAM is a technique used in deep learning to understand which regions of the input image are important for network prediction of specific categories, providing interpretability without affecting accuracy. The highlighted regions in the heatmaps are shown in red, and the weak regions are shown in blue. The red and blue marks represent the regions of strong and weak emphases, respectively^36^. Based on Grad-CAM, the key regions of IHC images of CCNT1 protein were obtained, which contribute more to protein subcellular localization prediction results. At the same time, in order to gain a deeper understanding of the impact of different dimensional features on prediction results, we analyzed the key regions for feature extraction in different convolutional layers^37^. It was found that the range of consideration for 64 dimensional features was relatively large. When the dimension increased to 128 dimension, the focus area was more precise and the boundaries are clearer. However, as the dimension increased, the focus area tended to be more dispersed, which may be less accurate (Fig. 7). This result indicated that the 128 dimensional parameters we trained were more optimal and can better predict protein localization, which can better screen for localization changes and of great significance for the diagnosis of breast cancer.

It should be noted that among these proteins, ZNF500 had no expression value, and no expression correlation analysis was performed. But literature verification found that ZNF500 suppressed breast cancer cell proliferation, and enrichment analysis found that it was enriched in breast cancer related cell line MCF7. At the same time, in the analysis of potential mechanisms, the significantly co-expressed and interacting proteins and co-located and interacting ncRNAs of differentially localized proteins were extracted. The co-location of co-expressed interacting proteins has not been taken into account as many proteins lacked IHC images, making it impossible to predict their subcellular localization under disease status through models. On the other hand, the ncRNAs expression data of the corresponding samples in the protein expression profile were not available, so the correlation coefficient could not be calculated and the results of ncRNAs did not take co-expression into account.

Potential clinical application was also done to analyze the role of our results in diagnostic and therapeutic pathways of breast cancer. IHC images have been widely used as the gold standard for cancer diagnosis. Deep learning is renowned for its ability to correctly classify and segment images. Accurate and interpretable machine learning models are crucial for effective diagnosis and treatment planning. The interpretability of our models was explored and the key regions of IHC images were obtained base on Grad-CAM more accurately and quickly. Thus, our models could better predict protein localization and screen for localization changes more accurately and quickly, which is of great significance for the diagnosis of breast cancer. Moreover, potential clinical applications of differentially localized proteins in the diagnosis and treatment of breast cancer was analyzed based on the existing literature on breast cancer markers and targets. It was found that five differentially localized proteins we obtained and their interacting molecules have been used as therapeutic targets for breast cancer and played an important role in the diagnosis and treatment of breast cancer (CCNT1^18,38,39^, PRPF4^19^, RECQL4^20^, UTP6^39^, ZNF500^21^). The role of NSUN5 in the diagnosis and treatment of breast cancer has not been clearly studied, but studies have shown that the protein is closely related to the prognosis of hepatocellular carcinoma^40^. It may also play a role in the diagnosis and treatment of breast cancer.

In addition, two Phosphoproteome data corresponding to the two profiles were downloaded (PDC000121, PDC000174) to analyze whether the differentially localized proteins were modified and the relationship between the modification and breast cancer. By analyzing the expression of phosphorylated differentially localized proteins in disease and normal samples, it was found that RECQL4 protein was differentially downregulated in both sets, while NSUN5 protein was differentially upregulated in PDC000174. These two proteins were not differentially expressed in both proteome expression profiles, suggesting that the phosphorylation of these two proteins did not affect expression, but might have an effect on localization, which might play a role in breast cancer.

In particular, in order to verify whether the protein localization changes we identified were affected at the transcriptional level, the breast cancer gene expression data corresponding to samples of the protein expression profile was downloaded from the TCGA database. The gene (inter‐sample) correlation of 6985 mRNA-protein pairs in all patient samples was investigated through spearman correlation coefficient and T-test. In both analysis, significant *P* values in the T-test and low Spearman correlation coefficients were observed (Supplementary Fig. 1). It indicated that there was an inconsistency between mRNA and protein at the expression level^41^. It was worth noting that the differential expression analysis of transcriptome data showed that the differentially localized proteins we obtained were not differentially expressed, while most of them were also not differentially expressed in the protein expression profile. This indicated that our differential localization analysis can yield results that cannot be obtained by differential expression analysis.

At the same time, the expression of genes corresponding to the proteins at the single-cell level was also evaluated using 12 breast cancer-related datasets in the Tumor Immune Single Cell Hub 2 database (TISCH2; http://tisch.comp-genomics.org/; accessed on 7 July 2023). The TISCH2 is a resource of single-cell RNA-seq data from human and mouse tumors, which enables comprehensive characterization of gene expression in the tumor microenvironment across multiple cancer types^42^. As ZNF500 was not found in the database, only the remaining five genes were analyzed. It was found that most genes are highly expressed in immune cells, especially four genes (CCNT1, NSUN5, PRPF4, RECQL4) are highly expressed in proliferating T cells. CCNT1 and NSUN5 are highly expressed in malignant cells, while UTP6 is highly expressed in epithelial cells and fibroblasts (Supplementary Fig. 2). All results demonstrated that these genes have high levels of malignant and immune cells at mRNA levels, which may be the causes of immune microenvironment and tumor heterogeneity^43^.

Through the analysis of breast cancer pathway in the KEGG database, it was found that the interacting molecules and interaction relationships of differentially localized proteins we obtained were common in different subtypes.

Since only few protein immunohistochemistry image datasets are currently available, and even fewer data that contain both image and localization information, we used data from the HPA database. In order to find external datasets, we searched relevant literature on IHC image studies and found that IHC images used in these studies were all tumor tissue images and no normal tissue images. We downloaded the breast cancer IHC image dataset constructed by Gil et al. for analysis^44^ without the true location of proteins in these tumor tissue images. Therefore, 25 proteins with location information in the breast cancer cell line MCF7 in the SUBCELL BARCODE database were considered. Based on the model we constructed, the subcellular localization of proteins was predicted. It was found that the predicted localization of 19 proteins was consistent with the protein localization in the cell line. Due to the lack of information such as staining intensity level and quantity in the dataset, it is not possible to further filter the images, which may lead to inaccurate prediction results. However, our model still achieves good prediction results with an accuracy rate of 0.76, indicating that the model could be applied to external datasets. Further comparison with the localization information in normal breast tissue from HPA revealed that the predicted localization of 9 proteins in the 19 proteins was inconsistent with the localization in normal tissues (BIRC5, ESR1, GABARAP, IGFBP2, IGFBP5, MLH1, PGP, PTEN, YBX1). These proteins have been confirmed as breast cancer related, which might be caused by their localization changes (Supplementary Table 3). In addition, these proteins had not overlapping with the 332 proteins obtained from HPA, and can be used not only as independent validation sets for further model validation, but also to complement the results of HPA.

In addition, the HPA database lacked cancer subtype information, for which differentially localized proteins could not be used for the classification of breast cancer subtypes. If HPA could provide subtype information in the future, localization studies on breast cancer subtypes might be possible through our model. Our method is currently only used in breast cancer, which could be applied to other cancers in the future.

## Methods

**Fig. 8:**
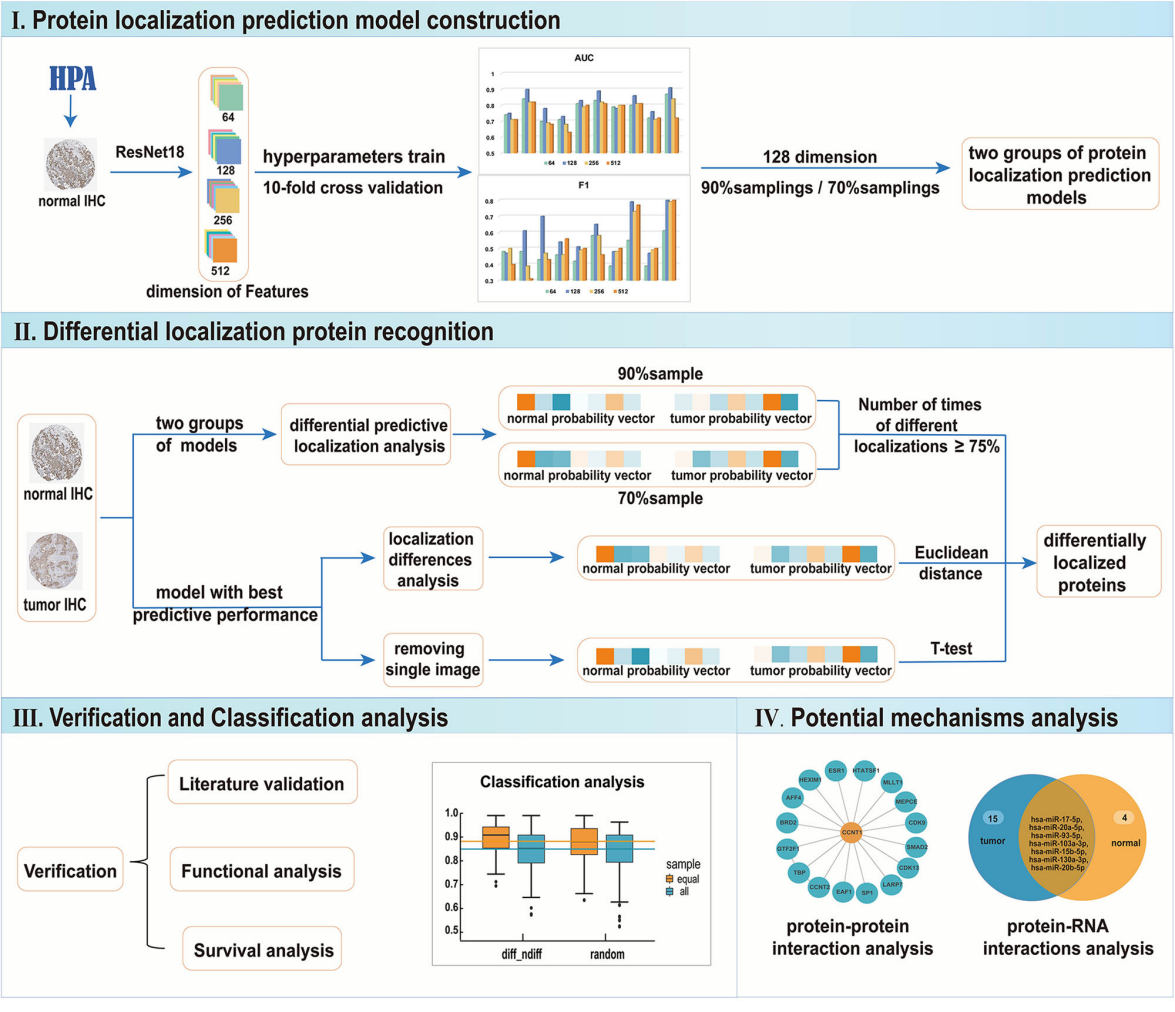
Flowchart. I. Construct and train the model based on the deep learning framework. II. Identification of differentially localized proteins in breast cancer. III. Verification of the relationship between differentially localized proteins and breast cancer and classification performance analysis of the proteins. IV. The relationship between localization changes and breast cancer was analyzed through potential mechanisms analysis.

Based on the deep learning model, the features of breast IHC images were analyzed and protein subcellular location prediction models were constructed to identify differentially located proteins in breast cancer. Meanwhile, their potential mechanism and relationship with breast cancer were analyzed. The detailed steps are shown in the flowchart (Fig. 8).

### Data

The IHC images of breast tissue were downloaded from the Human Protein Atlas database. The XML files of breast tissue images were obtained from the HPA based on the hpaXmlGet function in the R package “HPAanalysis”. Only Image whose staining intensity level is strong or moderate and quantity is higher than 75% was screened and downloaded for the subsequent research.

The subcellular localization information under normal conditions was downloaded from the HPA database. According to the hierarchical structure of organelles and the number of proteins of each category, all subcellular location labels in the data were classified into the following seven categories: Cytoplasm, Endoplasmic reticulum, Golgi apparatus, Mitochondria, Plasma membrane, Nuclear, Vesicles. In order to ensure the reliability of labels, only images whose label confidence level was ‘enhanced’ in the cell atlas were retained. Subsequently, the images of proteins with both normal and disease images were extracted. Considering that using only one image to predict localization is not accurate or reliable, proteins with only one image in the screening results were removed. Finally, 3375 images of 332 proteins were obtained (Table 2).

**Table 2:** Label distribution in the dataset

Label	Protein	Normal images	Tumor images
Cytoplasm	72	179	537
Endoplasmic reticulum	12	30	105
Golgi apparatus	20	50	145
Mitochondria	46	108	359
Plasma membrane	19	47	126
Nuclear	170	396	1342
Vesicles	24	59	187
Total	332	792	258

The protein expression data and corresponding clinical information in Breast Invasive Carcinoma and paracancerous tissue were downloaded from the Clinical Proteomic Tumor Analysis Consortium (CPTAC; https://proteomic.datacommons.cancer.gov/pdc/; accessed on 7 December 2022) database for subsequent analysis. Then, the data was preprocessed by deleting proteins with over 30% missing values and the imputation of the missing values in the remaining proteins was done based on the K-Nearest Neighbor (KNN) method. Here two sets of protein expression profiles were downloaded and organized: PDC000120 and PDC000173. The former compiled expression data for 9124 proteins from 143 samples, while the latter obtained 8790 protein expression data from 108 samples (Supplementary Table 4).

### Construction of protein localization prediction models

Based on the deep learning framework, protein subcellular localization prediction models for breast cancer were built using features extracted from IHC images^10^ (Supplementary Fig. 3).

The ResNet_18 model was adopted to extract features from IHC images. ResNet_18 is a classic and effective deep convolutional neural network model with excellent feature extraction and classification capabilities, which can be applied to computer vision tasks such as image classification and object detection. The ResNet18 model has four stages, each with 2 BasicBlocks. Each stage extracts features in different dimensions, namely 64, 128, 256, and 512 dimensions. The image information described by features of different dimensions is different. According to previous studies on interpreting the feature map of CNNs, the low-level features describe detailed information, e.g., textures, colors and edges, while high-level features, which are more abstract, capture more position-independent semantic information^45^. In order to investigate the impact of features of different dimensions, the four types of features with different dimensions were used to construct the models separately and the performance was compared.

The features of images of normal samples were incorporated into the Transformer model to aggregate the image features, and predict protein subcellular localization. The Transformer is a model that uses attention, a concept that helped improve the performance of neural machine translation applications, to boost the speed with which these models can be trained^16^. It can accept multiple input feature vectors, comprehensively consider all vectors and find useful information from them. The image features obtained in the previous step were input into the model for feature aggregation, and finally, each protein obtained a predicted probability vector. The value in each column of the vector represents the probability of the protein located to the position.

To improve the efficiency of the model, the hyperparameters were trained. Hyperparameters in the model include the dimension of the input features, the depth of the model, the number of heads, the number of hidden neurons in the feedforward layer, etc. Here the dimension of the input features was mainly trained and evaluated. For other parameters, referring to the Imploc model, the depth of the model was set to 4, the number of heads was set to 6, and the number of hidden neurons in the feed forward layer was set to four times of the feature dimension. In order to determine the most appropriate feature dimension, the four types of features with different dimensions were used to construct the models, respectively.

Ten percent of the proteins were randomly selected as the test set, and the remaining proteins were used to train the models by 10-fold cross validation. The proteins were redivided into 10 parts. Each time, one of the 10 parts was used as the validation set, and the rest were used as the training set. After 10 training sessions, 10 models were obtained. To evaluate the effectiveness of the models, the downloaded localization information from HPA was used as known subcellular localization and the AUC value and F1 score$\left(1\right)$ were calculated.

1 $$F1=2\cdot \left({precision}\cdot {recall}\right)/\left({precision}+{recall}\right)$$

Where ${precision}$ refers to the proportion of positive samples in the positive cases determined by the classifier, and ${recall}$ refers to the proportion of predicted positive cases to the total positive cases.

Comparing the results of the four dimensions, the dimension of the feature with the best prediction effect was used as the hyperparameter of the model.

At the same time, the probability of a protein being located at each position was predicted. When the probability of the protein being localized to that position is greater than the threshold we set, the protein was localized to that position. Multiple localizations may be obtained for each protein. Multiple localizations may be obtained for each protein and 6 thresholds were considered (0.3, 0.4, 0.5, 0.6, 0.7, 0.8). Comparing the results of different dimensions and thresholds, the effectiveness of the models was evaluated.

Using the optimal hyperparameters, the images were retrained with all 10 parts as training sets to obtain final models. To ensure the reliability and reproducibilityof the results, two sampling methods that divide the training set and the test set were applied to construct the protein localization prediction models. Ninety percent and seventy percent of the samples were randomly selected as the training set, and the remaining samples as the test set to evaluate the effectiveness of models. Both methods were repeated 10 times, resulting in two sets of models with 10 in each group.

To further evaluate the predictive performance of our models, two existing methods for protein subcellular localization prediction based on convolutional neural networks, AnnoFly and Imploc, were used. Based on IHC images of normal breast tissue, protein subcellular localization was predicted and the results were compared with those of our models. To ensure the accuracy of the results, the same data and sampling methods were used for the three models. The AUC value and F1 values were calculated and predictive effectiveness of our models were evaluated.

### Identification of differentially localized proteins

Based on the constructed subcellular location prediction models, the localizations of proteins were mainly predicted based on the following three aspects:

First, identification of stable differentially predictive localized proteins. The normal images and cancer images were input into the models separately to predict subcellular localization, and two prediction probability vectors were obtained for each protein (${P}_{n},\,{P}_{c}$). Since the subcellular localizations downloaded from the HPA database were classified into seven categories, the probability vector was a 7-dimensional vector.

2 $${P}_{c}={\left[{p}_{c1}\,{p}_{c2}\cdots {p}_{c7}\right]}^{T}$$

3 $${P}_{n}={\left[{p}_{n1}\,{p}_{n2}\cdots {p}_{n7}\right]}^{T}$$

Each element of the vector represented the probability that the protein will be located on that location label. If the probability was greater than threshold ($p$) the protein was predicted to be located at that location$\left(4\right)$.

4 $${L}_{P}=\left\{{L}_{i}|\,{p}_{i}\, > \,p\right\}\,i\in \left[1,7\right]$$

Where ${L}_{P}$ is the localization of the protein predicted by the model, ${L}_{i}$ is the label of the i-th position, and ${p}_{i}$ is the probability of positioning to the i-th position.

For the 20 models obtained above, the proteins with different prediction results in tumor and normal conditions were extracted, respectively. For each set of models, the number of times that the predicted location of each protein was different in the results was counted, and proteins whose number was higher than the upper quartile were extracted. The results of the two groups were intersected to obtain proteins with stable differentially predictive localization$\left(5\right)$.

5 $${P}_{s}=\left\{P | {P}_{C70} \ge Q\, \& \,{P}_{C90} \ge Q\right\},\,Q=\left[0.75 * \left(n+1\right)\right]$$

Where ${P}_{s}$ represents proteins with stable differentially predicted localization, ${{{{\rm{Q}}}}}$ is the value rounded down to the upper quartile of the counts, and ${P}_{C70}/{P}_{C90}$ is the count of times for proteins with different prediction results in all models obtained using 70% or 90% random sampling methods, respectively.

Second, identification of proteins with maximum localization differences. The model with the best predictive performance among all models was selected for the analysis. For each protein, the Euclidean distance between the two probability vectors in tumor and normal conditions (${P}_{n},\,{P}_{c}$) was calculated and the top 5% of the proteins with the farthest distance were obtained as the proteins with the largest localization difference.

Third, identification of proteins whose predicted results were not affected by the removal of a single image. For each protein, in order to exclude the influence of a single image on the prediction results, one image was deleted and the localization of the protein was predicted based on other images of the protein. Two probability vectors were obtained: the normal vector and the cancer vector. A T-test was performed on the prediction vector of removing a single image and the prediction vector of all images corresponding to the same protein, and proteins with insignificant differences in both disease and normal conditions were extracted (*P* < 0.05).

The intersection of the results of the above three steps was extracted as the stable differentially localized protein.

### Verification of differentially localized proteins

The relationship between differentially localized proteins and breast cancer was verified in the following aspects:

First, literature validation. Literature related to differentially localized proteins and breast cancer in the PubMed database were browsed to analyze the role of these proteins in the progression of breast cancer.

Second, functional analysis. Based on the enrichr function in the R package “enrichR”, Gene Ontology (GO; http://geneontology.org/; current release June 11, 2023) and Kyoto Encyclopedia of Genes and Genomes (KEGG; https://www.kegg.jp/; version: 107.0) analysis were performed on proteins. Biological processes and pathways with *P* values less than 0.05 were extracted as significantly enriched functions to analyze the association between the proteins and breast cancer.

Third, survival analysis. The survival data of the corresponding samples in the CPTAC profiles were downloaded from The Cancer Genome Atlas (TCGA; https://www.cancer.gov/ccg/research/genome-sequencing/tcga; accessed on April 2022) database, and survival analysis was done through the R package “survival”. Overall survival was defined as the time from the date of initial surgical resection to the date of death or last contact. Survival curves were drawn using the Kaplan–Meier method and were statistically compared using the log-rank test^46^. Based on the results of survival analysis (*p* < 0.05), the relationship between proteins and prognosis of breast cancer was discussed.

### Classification performance analysis

In order to evaluate the effectiveness of the proteins we obtained in distinguishing breast cancer from normal samples, the expression of differential localization proteins in tumor and normal samples was compared and a classifier was constructed for classification analysis.

Based on two sets of protein expression profiles downloaded from the CPTAC database, it was investigated that whether there were differences in the expression of differentially localized proteins. T-tests and foldchange analysis were adopted on two sets of data respectively, and proteins with *P* < 0.05 and $\left|{\log }_{2}{{{{\rm{foldchange}}}}}\right| > 1$ were identified as differentially expressed proteins.

A random forest model was adopted to construct a classifier based on the expression value of proteins to classify cancer and normal samples. Based on R package “randomForest”, the model was constructed and two important parameters of random forest, the number of decision trees in the model, ntree, and the number of variables contained in each decision tree, mtry, were set to 500 and ${\log }_{2}{{{{\rm{N}}}}}$, where N is the number of samples. The classification performance of the model was evaluated through the Leave One Out Cross Validation method. Then, an equal number of non-differentially localized proteins were randomly selected to construct a classifier, and the classification performance of the obtained proteins was compared with other proteins. Then, classifiers based on non-differentially localized proteins were constructed. Comparing the classification performance of the two groups of proteins, the ability of the obtained proteins to distinguish between normal and cancer samples was evaluated.

### Analysis of breast cancer potential mechanisms

The breast cancer potential mechanisms were investigated by extracting the co-expressed or co-located proteins and ncRNAs interacting with differentially localized proteins.

Based on the protein expression profiles of breast tissue downloaded from the CPTAC database, the Pearson correlation coefficients between differentially localized proteins and other proteins in normal and cancer samples were calculated, respectively. Proteins whose correlation coefficient >0.3 and *P* < 0.05 were extracted as co-expressed proteins. Then, the interaction data of the differentially localized proteins was downloaded in the STRING (https://cn.string-db.org/; version: 11.5) database and the interaction relationships with scores higher than 700 were extracted. By taking the intersection of the two, the significantly co-expressed and interacting proteins of differentially localized proteins were obtained. Finally, two groups of proteins under normal and cancer conditions were obtained and two groups of modules were constructed. In order to study the functional changes of differentially localized proteins between tumor and normal conditions, the functions of the protein under one condition were defined as the functions of all proteins in the interaction module under corresponding condition. GO and KEGG enrichment analysis were conducted for the proteins in the modules based on R package “clusterProfiler”. Biological processes and pathways with *P* value less than 0.05 were taken as the significantly enriched functions and pathways, which were used to study the changes of functions caused by localization changes and their impact on breast cancer.

The interaction relationships between RNA and protein were downloaded in the RNAInter database and non-coding RNA who interacts with the differentially localized proteins was extracted. Then, the positioning information of these RNAs was retrieved from the RNALocate database. Finally, ncRNAs with the same localization as the differentially localized proteins under both cancer and normal conditions were extracted. These ncRNAs were recognized as co-located RNAs of differentially localized proteins, which undergo localization changes together with the proteins. Their interaction might affect the localization of proteins, and thereby affecting their functions, which is of great significance for the occurrence of cancer.

### Statistics and Reproducibility

For all hypothesis testing, we used a standard threshold of *p* < 0.05 to assign significance. To improve the efficiency of the model, two hyperparameters were trained and two sampling methods were adopted. Full details were provided in the results and methods sections. The source data and code used in this research have been uploaded to the GitHub and Zenodo^47^ and can be accessed online at https://github.com/wendyliwan/protein-localization-prediction-models and 10.5281/zenodo.12139567.

### Reporting summary

Further information on research design is available in the Nature Portfolio Reporting Summary linked to this article.

### Supplementary information

Supplementary Information

Reporting Summary

## Data Availability

The datasets analysed during the current study are available in the Human Protein Atlas (HPA) database [https://www.proteinatlas.org/] and the Clinical Proteomic Tumor Analysis Consortium (CPTAC) database [https://proteomic.datacommons.cancer.gov/pdc/] with accession numbers PDC000120 and PDC000173. The external datasets can be downloaded from [http://bliss.gpec.ubc.ca] by navigating to 02-008 for the BCCA cohort. The statistical data for graphs and charts used in this research is available via 10.5281/zenodo.12139567. All other data are available from the corresponding author upon reasonable request.

## Code availability

The code used in this research has been deposited at 10.5281/zenodo.12139567 and at https://github.com/wendyliwan/protein-localization-prediction-models.
