# Supplementary material for: Differentially localized protein identification for breast cancer based on deep learning in immunohistochemical images
Source: Commun Biol. 2024 Aug 2;7:935. doi: 10.1038/s42003-024-06548-0 (PMC11297317; doi:10.1038/s42003-024-06548-0)
Supplement: Supplementary file 1 — Supplementary Information [file 42003_2024_6548_MOESM1_ESM.pdf]

Differentially Localized Protein Identification for Breast Cancer Based on Deep Learning in Immunohistochemical Images

Supplementary information

Supplementary Figure 1: mRNA-protein correlation analysis.

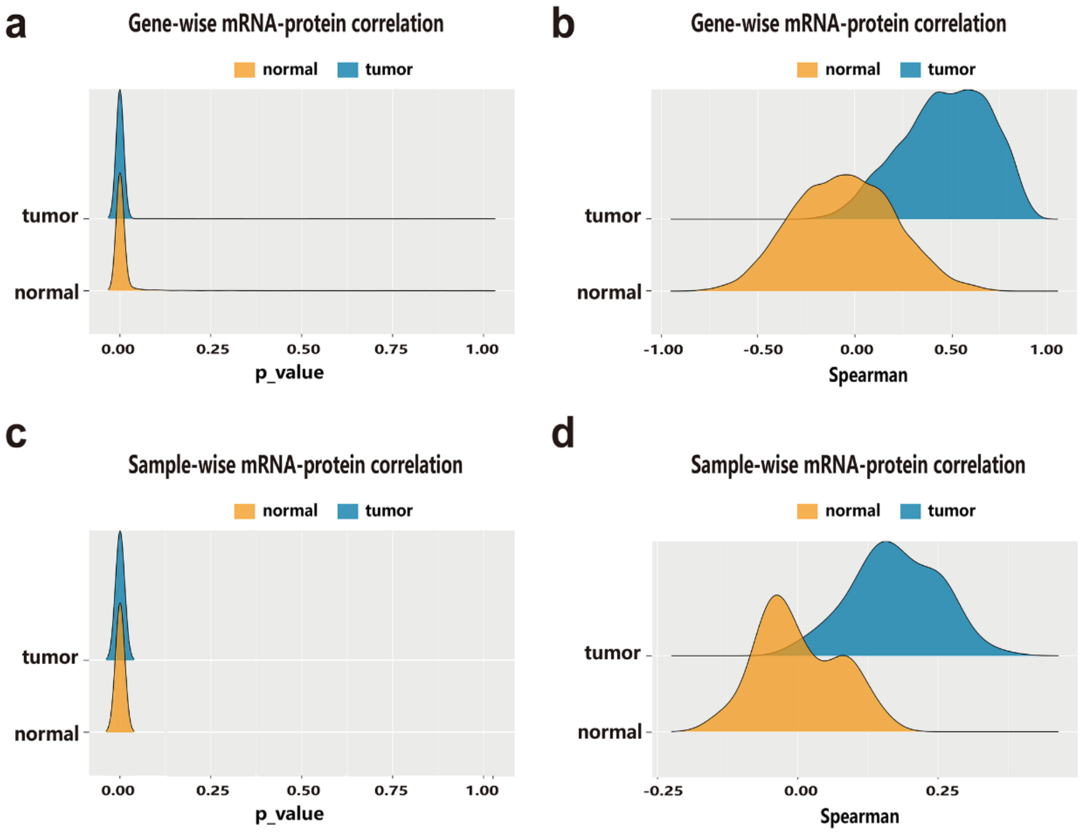

(a) P-values in the T-test of gene-wise mRNA-protein correlation analysis; (b) Spearman correlation coefficients of gene-wise mRNA-protein correlation analysis; (c) P-values in the T-test of sample-wise mRNA-protein correlation analysis; (d) Spearman correlation coefficients of sample-wise mRNA-protein correlation analysis.

Supplementary Figure 2: The expression of genes at the single-cell level.

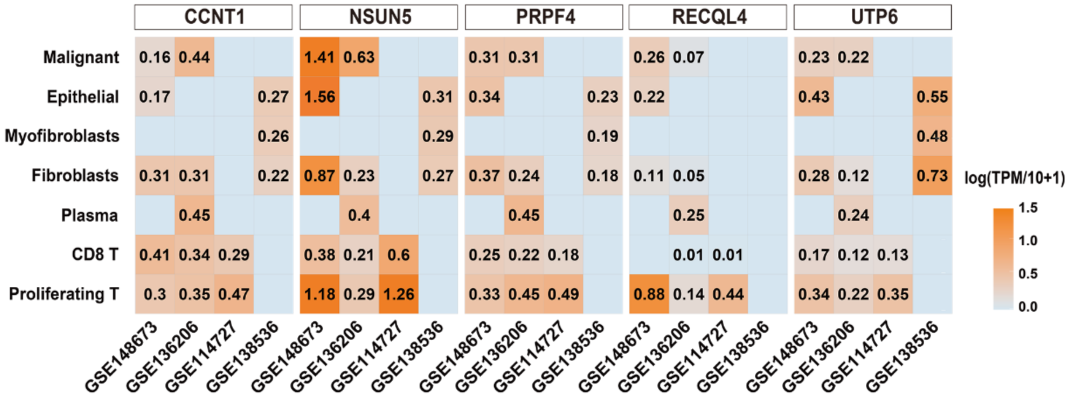

Only the four breast cancer datasets with the most significant expression differences are shown here.

Supplementary Figure 3: Protein localization prediction model.

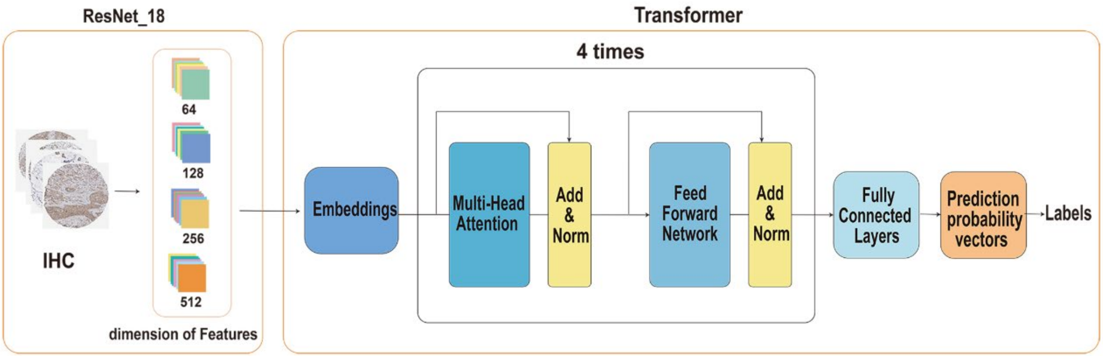

Features with different dimensions were extracted from IHC images based on ResNet_18 model and were incorporated into the Transformer model to construct the protein localization prediction model.

Supplementary Table 1: AUC value and F1 score of the predicted results of two sets of models.

	90%sample		70%sample	
	AUC	F1	AUC	F1
fold1	0.85	0.65	0.95	0.81
fold2	0.92	0.76	0.9	0.81
fold3	0.93	0.73	0.93	0.77
fold4	0.88	0.79	0.94	0.83
fold6	0.94	0.79	0.92	0.82
fold7	0.84	0.62	0.9	0.75
fold8	0.91	0.71	0.85	0.67
fold9	0.94	0.82	0.94	0.78
fold10	0.98	0.85	0.93	0.85
mean	0.92	0.76	0.92	0.81

Supplementary Table 2: Co-expressed interacting proteins of differentially localized proteins.

Protein	Normal Modules	Tumor Modules
CCNT1	AFF1; CCNB1; CCNK; CCNT2; CDK13; CDK4; CDK9; CTDP1; ELL; ERCC3; GTF2F1; HEXIM1; HTATSF1; IWS1; MNAT1; POLR2F; SMAD2; TAF11	AFF4; BRD2; CCNT2; CDK13; CDK9; EAF1; ESR1; GTF2F1; HEXIM1; HTATSF1; LARP7; MEPCE; MLLT1; SMAD2; SP1; TBP.
NSUN5	NULL	DIMT1; RRP1B; WDR3.
PRPF4	SNRPA1	BUD13; DDX23; DHX16; EFTUD2; ELAVL1; GPKOW; IK; LSM2; LSM4; LSM8; PHF5A; PPIH; PRPF18; PRPF19; PRPF3; PRPF31; PRPF38A; PRPF4B; PRPF8; RBM25; RBM42; RNF113A; RP9; SART1; SF3A1; SF3A2; SF3A3; SF3B1; SF3B2; SF3B3; SF3B4; SF3B6; SMU1; SNIP1; SNRNP200; SNRNP27; SNRNP40; SNRPA1; SNRPC; SNRPD2; SNRPD3; SNRPF; SNRPG; TFIP11; TXNL4A; USP39; ZGPAT.
RECQL4	FANCD2	ATR; CHTF18; FANCD2; FEN1; GINS4; KIFC1; MCM2; MCM3; MCM4; MCM5; MCM6; MCM7; MDC1; RAD18; TOP2A; UBR2; WDHD1.
UTP6	BMS1; DDX55; DKC1; DNTTIP2;FBL; MYBBP1A; NCL; NOLC1; NOP56; PES1; RRP36; WDR43	AATF; ABT1; BMS1; BOP1; BRIX1; BYSL; CEBPZ; CMSS1; DCAF13; DDX10; DDX18; DDX24; DDX27; DDX28; DDX31; DDX47; DDX49; DDX51; DDX52; DDX54; DDX55; DDX56; DHX33; DHX37; DIMT1; DKC1; DNTTIP2; EBNA1BP2; EIF4A3; EMG1; ESF1; EXOSC10; EXOSC2; EXOSC3; EXOSC4; EXOSC5; EXOSC6; EXOSC7; EXOSC8; EXOSC9; FBL; FCF1; FTSJ3; GNL2; GNL3; GNL3L; GRWD1; GTPBP4; HEATR1; IMP3; IMP4; KRI1; KRR1; LSG1; LTV1; LYAR; MAK16; MPHOSPH10; MRPS7; MRTO4; MYBBP1A; NAT10; NCL; NGDN; NIFK; NIP7; NMD3; NOB1; NOC2L; NOC3L; NOC4L; NOL10; NOL11; NOL12; NOL6; NOL7; NOLC1; NOM1; NOP14; NOP2; NOP56; NOP58; NOP9; NSA2; PAK1IP1; PDCD11; PES1; PNO1; POLR1A; POLR1B; POLR1C; POLR1E; PPAN; PRC1; PRPF31; PWP2; RBM19; RBM28; RBM34; RCL1; RPF1; RPF2; RPL7L1; RPP38; RPS15A; RPS16; RPS18; RPS24; RPS4X; RRP1; RRP12; RRP15; RRP1B; RRP36; RRP7A; RRP8; RRP9; RRS1; RSL1D1; RSL24D1; SDAD1; SURF6; TBL3; TEX10; TFB2M; TRMT6; TRMT61A; TSR1; TWISTNB; UTP14A; UTP15; UTP18; UTP20; UTP23; UTP3; WDR12; WDR18; WDR3; WDR36 ;WDR4 ;WDR43; WDR46; WDR74; WDR75.

Supplementary Table 3: Positioning prediction results and literature verification of the proteins.

	Predicted localization	Normal localization	localization in MCF7	Literature validation
BIRC5	Nuclear	Cytoplasm	Nuclear	PMID：35331169[1]
ESR1	Mitochondria	Vesicles	Secretory; Nuclear; Mitochondria; Cytoplasm	PMID：31318440[2]
GABARAP	Cytoplasm	Vesicles	Cytoplasm	PMID：33591943[3]
IGFBP2	Nuclear	Endoplasmic reticulum	Secretory; Nuclear; Cytoplasm; Mitochondria	PMID：37436978[4]
IGFBP5	Cytoplasm	Golgi apparatus; Vesicles	Nuclear; Secretory; Cytoplasm; Mitochondria	PMID：36093095[5]
MLH1	Cytoplasm	Nuclear	Nuclear; Secretory; Cytoplasm; Mitochondria	PMID：30149959[6]
PGP	Cytoplasm	Nuclear	Cytoplasm	PMID：37741091[7]
PTEN	Cytoplasm	Nuclear	Cytoplasm	PMID：30539826[8]
YBX1	Nuclear	Cytoplasm	Nuclear; Secretory; Cytoplasm; Mitochondria	PMID：25957686[9]

Supplementary Table 4: The statistics of protein expression data.

Data ID	PDC000120	PDC000173
**Patient(n)**	143	108
tumor	125	105
normal	18	3
**Age, years**		
range	30-90	30-89
median	62	59
**Sex(n)**		
female	130	103
male	0	2
not reported	13	3
**Tumor stage**		
Stage I	4	10
Stage II	75	66
Stage IIIA	34	26
Stage IV	0	2
Not Reported	28	4

1 Fäldt Beding, A., Larsson, P., Helou, K., Einbeigi, Z. & Parris, T. Pan-cancer analysis identifies BIRC5 as a prognostic biomarker. *BMC cancer* **22**, 322 (2022). https://doi.org:10.1186/s12885-022-09371-0

2 Dustin, D., Gu, G. & Fuqua, S. ESR1 mutations in breast cancer. *Cancer* **125**, 3714-3728 (2019). https://doi.org:10.1002/cncr.32345

3 Liu, Y. *et al.* GABARAP suppresses EMT and breast cancer progression via the AKT/mTOR signaling pathway. *Aging* **13**, 5858-5874 (2021). https://doi.org:10.18632/aging.202510

4 Conway, J. *et al.* IGFBP2 secretion by mammary adipocytes limits breast cancer invasion. *Science advances* **9**, eadg1840 (2023). https://doi.org:10.1126/sciadv.adg1840

5 Dittmer, J. Biological effects and regulation of IGFBP5 in breast cancer. *Frontiers in endocrinology* **13**, 983793 (2022). https://doi.org:10.3389/fendo.2022.983793

6 Malik, S. *et al.* Expressional analysis of MLH1 and MSH2 in breast cancer. *Current problems in cancer* **43**, 97-105 (2019). https://doi.org:10.1016/j.currproblcancer.2018.08.001

7 Szebényi, K. *et al.* Effective targeting of breast cancer by the inhibition of P-glycoprotein mediated removal of toxic lipid peroxidation byproducts from drug tolerant persister cells. *Drug resistance updates : reviews and commentaries in antimicrobial and anticancer chemotherapy* **71**, 101007 (2023). https://doi.org:10.1016/j.drup.2023.101007

8 Li, K., Li, G., Sun, L. & Li, X. PTEN and SHIP: Impact on lymphatic metastasis in breast cancer. *Journal of cancer research and therapeutics* **14**, S937-S941 (2018). https://doi.org:10.4103/0973-1482.193894

9 Goodarzi, H. *et al.* Endogenous tRNA-Derived Fragments Suppress Breast Cancer Progression via YBX1 Displacement. *Cell* **161**, 790-802 (2015). https://doi.org:10.1016/j.cell.2015.02.053
